# Bone Marrow Microenvironment Interplay and Current Clinical Practice in Multiple Myeloma: A Review of the Balkan Myeloma Study Group

**DOI:** 10.3390/jcm10173940

**Published:** 2021-08-31

**Authors:** Jelena Bila, Eirini Katodritou, Margarita Guenova, Sandra Basic-Kinda, Daniel Coriu, Milena Dapcevic, Lejla Ibricevic-Balic, Arben Ivanaj, Oliver Karanfilski, Samo Zver, Meral Beksac, Evangelos Terpos, Meletios Athanassios Dimopoulos

**Affiliations:** 1 Clinic of Hematology, Clinical Center of Serbia, Faculty of Medicine, University of Belgrade, 11000 Belgrade, Serbia; 2 Department of Hematology, Theagenio Cancer Hospital, 54639 Thessaloniki, Greece; eirinikatodritou@gmail.com; 3 Laboratory of Haematopathology and Immunology, National Specialised Hospital for Active Treatment of Haematological Diseases, 1756 Sofia, Bulgaria; margenova@gmail.com; 4 Divison of Hematology, Department of Internal Medicine, University Hospital Centre Zagreb, School of Medicine, University of Zagreb, 10000 Zagreb, Croatia; sandra.kinda@gmail.com; 5 Centre of Hematology and Bone Marrow Transplant, “Fundeni” Clinical Institute, “Carol Davila” University of Medicine and Pharmacy, 022328 Bucharest, Romania; daniel_coriu@yahoo.com; 6 Division of Hematology, Clinical Center of Montenegro, Podgorica 81000, Montenegro; milena.dapcevic@yahoo.com; 7 Clinic of Hematology, University Clinical Center of Sarajevo, 71000 Sarajevo, Bosnia and Herzegovina; lejla99@hotmail.com; 8 Department of Hematology, University Medical Center “Mother Teresa”, 1001 Tirana, Albania; aivanaj@yahoo.fr; 9 University Clinic of Hematology, Faculty of Medicine, University of Skopje, 1000 Skopje, North Macedonia; karanfilski@yahoo.com; 10 Department of Hematology, University Medical Centre Ljubljana, 1000 Ljubljana, Slovenia; samo.zver@kclj.si; 11 Department of Hematology, Tissue Typing Laboratory and Donor Registry, Faculty of Medicine, University of Ankara, Ankara 06590, Turkey; meral.beksac@medicine.ankara.edu.tr; 12 Department of Clinical Therapeutics, Alexandra General Hospital, School of Medicine, National and Kapodistrian University of Athens, 11528 Athens, Greece; eterpos@med.uoa.gr (E.T.); mdimop@med.uoa.gr (M.A.D.)

**Keywords:** multiple myeloma, bone marrow microenvironment, treatment

## Abstract

The course of multiple myeloma (MM) is influenced by a variety of factors, including the specificity of the tumour microenvironment (TME). The aim of this review is to provide insight into the interplay of treatment modalities used in the current clinical practice and TME. Bortezomib-based triplets are the standard for MM first-line treatment. Bortezomib is a proteasome inhibitor (PI) which inhibits the nuclear factor kappa B (NF-κB) pathway. However, bortezomib is decreasing the expression of chemokine receptor CXCR4 as well, possibly leading to the escape of extramedullary disease. Immunomodulatory drugs (IMiDs), lenalidomide, and pomalidomide downregulate regulatory T cells (Tregs). Daratumumab, anti-cluster of differentiation 38 (anti-CD38) monoclonal antibody (MoAb), downregulates Tregs CD38+. Bisphosphonates inhibit osteoclasts and angiogenesis. Sustained suppression of bone resorption characterises the activity of MoAb denosumab. The plerixafor, used in the process of stem cell mobilisation and harvesting, block the interaction of chemokine receptors CXCR4-CXCL12, leading to disruption of MM cells’ interaction with the TME, and mobilisation into the circulation. The introduction of several T-cell-based immunotherapeutic modalities, such as chimeric-antigen-receptor-transduced T cells (CAR T cells) and bispecific antibodies, represents a new perspective in MM treatment affecting TME immune evasion. The optimal treatment approach to MM patients should be adjusted to all aspects of the individual profile including the TME niche.

## 1. Introduction

The change in the multiple myeloma (MM) landscape, brought forth by novel regimens, has been revolutionary, also improving diagnostics over the last two decades [1,2,3]. Characterised by estimated annual incidence in Europe of 4.5–6.0 new patients per 100.000 inhabitants, MM is the second most common haematological malignancy [4]. Still, despite the possibility to achieve well-controlled chronicity, MM generally remains an uncurable disease [5]. The course of the disease is significantly influenced by a variety of clinical, laboratory, and genetic characteristics, including the specificity of the tumour microenvironment (TME) in the bone marrow (BM) milieu [6,7].

In the view of internationally recognised recommendations such as EHA-ESMO clinical practice guidelines for MM diagnosis, treatment, and follow-up [5], the aim of this review is to provide insight into the interplay of treatment modalities used in the current clinical practice and TME. 

## 2. Treatment Modalities in the Current Clinical Practice and TME Interplay

### 2.1. Proteasome Inhibitors (PIs)

Bortezomib is the first-in-class proteasome inhibitor, which, along with immunomodulatory drugs (IMiDs), has greatly changed the course of this disease [1]. Currently, the standard of care for the first-line treatment in MM are bortezomib-based triplets and quadruplets [5]. As an inhibitor of the NF-κB pathway, bortezomib induces apoptosis of MM cells and osteoclasts, inhibits pro mm cytokines, natural killer (NK) cell’s activity, and causes the differentiation of mesenchymal stem cells (MSCs) from osteoblasts [8,9,10]. Additionally, transcription of hypoxia-inducible factor-1alfa (HIF-1alfa) is suppressed by bortezomib, consequently leading to the inhibition of angiogenesis by decreasing the vascular endothelial growth factor (VEGF) [11]. Bortezomib overcomes cell adhesion-mediated drug resistance (CAM-DR) by inhibiting very late angine-4 (VLA-4) and expresses synergistic effect with various drugs in co-culturing human myeloma cell lines with bone marrow stromal cells [10,12]. Notably, one of the bortezomib effects is bone remodelling based on enhanced osteogenesis induced by the inhibition of receptor activator of nuclear factor kappa-B ligand (RANKL) and Dickkopf-1 (DKK-1), and activation of osteoblasts [13]. Bortezomib also decreases chemokine receptor CXCR4 expression, possibly leading to the escape of extramedullary disease [14]. Paradoxically, PIs also may induce the accumulation of pro-inflammatory macrophages, consequently leading to the MM cell survival and progression of the disease [15]. The resistance to bortezomib is expressed more often in MM cells characterised with immature immunological profiles, lacking CD138 expression [16].

Carfilzomib is a selective, second-generation irreversible PI. The therapeutic activity of carfilzomib is based on induction of unfolded protein stress response, inhibition of NF-κB activity, induction of NK cells activity, and bone remodelling with impact to TME as well [17]. Carfilzomib is characterised by structural differences in comparison to the bortezomib, and the ability to overcome bortezomib resistance, promoting deeper and more sustained proteasome inhibition [18].

First-in-class orally administered PI, ixazomib is characterised with the same structural class and activity as bortezomib, decreasing NF-κB signalling, followed by reduced osteoclastogenesis and enhanced differentiation of osteoblasts from mesenchymal stem cells (MSCs), and osteoblast’s activity [19].

### 2.2. Immunomodulatory Drugs (IMiDs) 

Since the introduction of alkylating agents in the MM treatment, the first drug that changed the course of the disease was thalidomide through multiple modes of action, e.g., antiangiogenesis induced by suppressed VEGF gene, accompanied with various immunomodulatory and anti-inflammatory effects [20,21]. Regarding interaction with TME, IMiDs are characterised, besides the anti-angiogenic effect, with pro-apoptotic activity, enhanced activity of T- and NK cells, as well as downregulation of TME’s cytokines, and inhibition of bone resorption [9]. One of the mechanisms of action of lenalidomide is inhibition of VEGF-induced phosphoinositide 3-kinase (PI3K)/Akt mTOR pathway and HIF-1alfa expression on endothelial cells [22].

Possible resistance for IMiDs in MM patients may be induced by decreased expression in the IKAROS zinc finger 1 (IKZF1) family of proteins, due to interactions between MM cells and bone marrow stromal cells (BMSCs) [23]. On the other hand, within combos of bortezomib-plus-IMiDs, the activity of IMiDs is enhanced due to the effects of bortezomib, as the release of CAM-DR and elevated expression of IKZF1 [7]. Due to the high expression of IKZF1, IMiDs affect dormant MM cells in TME, with proven efficacy of lenalidomide for immature MM cells, in comparison to the PIs affecting more mature MM cells [24]. One of the effects of lenalidomide and pomalidomide is the downregulation of regulatory T cells (Tregs). The function of regulatory T cells (Tregs) is suppressed by IMiDs, based on downregulation of the Foxp3 gene’s expression [25]. Interestingly, a similar effect on Tregs characterises treatment with low-dose cyclophosphamide. The addition of low-dose oral cyclophosphamide potentially may overcome refractoriness to lenalidomide [26].

Due to suppression of programmed death (PD)-1 antigen on T and NK cells by IMiDs in general, and PD-ligand-1 (PDL-1) in MM cells by lenalidomide, next-generation IMiDs such as lenalidomide and pomalidomide represent good partners of monoclonal antibodies (MoAbs) in MM treatment promoting antibody-dependent cellular cytotoxicity (ADCC) [27,28].

In comparison to PIs, IMiDs promote immune reconstitution. However, bortezomib and lenalidomide do not have the ability to suppress the activity of myeloid-derived suppressor cells (MDSCs) [7,25].

### 2.3. Monoclonal Antibodies (MoAbs)

Following the concept of directed immunochemotherapy, targeting specific antigens on the surface of malignant cells, extensive research in the MM field resulted in the introduction of monoclonal antibodies (MoAbs) [29]. 

Anti-cluster of differentiation 38 (anti-CD38) MoAbs are characterised with different therapeutic effects, e.g., ADCC, complement-dependent cytotoxicity (CDC), direct cytotoxicity, enhanced immune system, and different anti-TME effects such as inhibition of CAM-DR and induced bone remodelling [7,30].

First MoAb approved as monotherapy in MM patients who were heavily pre-treated with previous therapies containing PIs and IMiDs was daratumumab. Daratumumab binds CD38 surface antigen to malignant plasma cells [31]. Treatment with daratumumab induces downregulation of Tregs CD38+ cells and more pronounced immunosuppression in comparison to the Tregs CD38- [32]. The expression of CD38 is suppressed in the case of in vitro co-cultures with BMSCs, caused by the linkage of CD38 on MM cells with CD31 on BMSCs [33]. The therapeutic effect of daratumumab can be potentiated in combination with bortezomib due to inhibition of linkage CD38-CD31 by bortezomib, consequently leading to increased expression of CD38 target on MM cells. In contrast, due to the internalisation of CD38 in MM cells and consequent inhibition of adhesion to BMSCs by daratumumab, CAM-DR is released [34]. However, the activity of CAM-DR might be overcome by bortezomib inhibiting VLA-4.^12^ Interleukin 6 (IL6), as the main cytokine of MM growth, suppresses CD38 expression during the course of disease in relapsed/refractory MM patients, leading to the resistance to daratumumab [35].

The second MoAb in clinical practice is elotuzumab, which targets signalling lymphocytic activation molecule family member 7 (SLAMF7, CD319), a glycoprotein on the surface of MM cells [31]. The activity of both of MoAbs—daratumumab and elotuzumab—is independent of the stage of differentiation of MM cells. However, the condition of neoplastic hypoxia suppresses maturation of MM cells and expression of SLAMF7 and CD38, inducing the resistance to elotuzumab, daratumumab, or another anti-CD38 MoAb, isatuximab [7,36]. The combination of IMiDs and anti-PD-1 monoclonal antibody did not result in significant clinical benefit in MM patients, accompanied by reports of its notable toxicity [37,38].

Regarding the process of bone remodelling, the anti-RANKL MoAb denosumab acts by preventing skeletal events. Sustained suppression of osteoclastic bone resorption, based on inhibition of the interaction between receptor–activator of NF-κB ligand and its anchor receptor (RANKL-RANK) characterises the activity of denosumab [39].

### 2.4. Bisphosphonates

Amino-bisphosphonates are applied in MM patients as supportive therapy of bone disease due to inhibition of the osteoclasts and anti-angiogenic activities. Nitrogen-containing bisphosphonates bind to and inhibit the activity of farnesyl pyrophosphate synthase. The isoprenylation of proteins as Rab, Rac, and Rho, is inhibited, resulting in isolated osteoclast apoptosis before endocytosis within osteoclasts during the process of osteoclast-mediated bone mineral dissolution and matrix digestion [40]. Zoledronic acid, as a nitrogen-containing bisphosphonate, and anti-RANKL MoAb denosumab expressed comparable treatment results regarding skeletal events and progression-free survival of MM patients [41].

### 2.5. Autologous Stem Cell Transplantation (ASCT)

One of the major achievements in the treatment of multiple myeloma was the concept of high-dose therapy, followed by ASCT (HDT+ASCT), which is mainly caused by the ability to induce a better quality of response [7]. In the era of new drugs, HDT+ASCT retains its importance as the standard of care in fit MM patients usually <70 years [5,42]. Standard conditioning regimen is still Melphalan 200 mg/m^2^, non-specifically affecting MM cells, while autograft induces both the recovery of the myeloablative effect of HDT and improvement of microenvironment consisting of induced autograft’s MSCs differentiation to BMSCs, as well as different components of endosteal niche, e.g., osteoblasts, chondrocytes, or myocytes [43].

There is reported negative prognostic impact of the BM infiltration with M2 macrophages, secreting pro-tumoural immunosuppressive agents such as interleukin 10 (IL10), transforming growth factor β1 (TGF-β1), and Arginase-1, and pro-angiogenic factors such as VEGF and fibroblast growth factor 2 (FGF-2) on the treatment outcome and prognosis of patients treated with chemotherapy and ASCT [44].

Despite the introduction of highly effective new treatment modalities, HDT+ASCT remains the mainstay of first-line treatment in patients clinically eligible for such an approach, due to the ability to deepen the response and TME improvement, [45,46]. The benefit of ASCT has been re-evaluated by IFM 2009/DFCI phase 3 trial and EMN02 trial, confirming the superiority of treatment results, such as depth of response and progression-free survival, in the ASCT group, compared to the chemotherapy group, followed by similar overall survival for both groups [47,48]. 

## 3. Treatment Implications in the Current Clinical Practice

Traditionally, myeloma has been considered an uncurable disease. However, current advances in MM treatment based on the new modes of action of various treatment modalities resulted in the achievement of survival in 10–15% of MM patients, which is comparable to the average life expectancy of the general population [5,49].

A very complex individual MM patient’s prognostic profile, consisting of clinical condition presented by comorbidity and frailty indices; different prognostic scores such as International Staging System (ISS) and Revised ISS score (R-ISS) based on laboratory and prognostically significant chromosomal abnormalities (CA); specific CA of prognostic significance, e.g., abnormalities of chromosome 1, entities as double and triple hit myeloma; as well as delicate TME interplay, indicates treatment based on multi-drug combinations including PIs, IMiDs, MoAbs, with/without ASCT, in order to cover all the aspects of patient’s profile with synergistic therapeutic effect [50,51,52,53]. 

In an attempt to keep the balance between current recommendations and contemporary MM treatment, the question on the current clinical practice was raised. Concerning the modes of action and impact on TME, the activity of IMIDs and PIs is expressed on different levels of differentiation of MM cells [7]. The efficacy of PIs refers, regardless of CAs risk, predominantly on mature MM cells, inducing stress of endoplasmic reticulum, recovering hypoxia, bone remodelling, and improving ADCC [16,54,55]. In comparison to the PIs, IMiDs affect more immature MM cells, characterised with high expression of IKZF1 and absence of high-risk CAs, promote immune reconstitution by suppressing the activity of Tregs and expression of PD-1 and its ligand PDL-1, resulting in potentiated ADCC in combination with MoAbs [16,23,24,25,26,27,28]. The activity of anti CD38 and SLAMF-7 MoAbs is expressed in accordance with the extent of expression of CD38 and CD319 (SLAMF-7) antigens on MM cells, independently of the stage of differentiation [32,33,34]. However, during the progression of the disease, increased IL-6 suppresses CD38 expression, consequently leading to the possible lack of efficacy of anti-CD38 MoAbs, implicating preferable application in the early phase of the disease [35]. Based on these various, still complementing and synergistic pharmacological effects, different combinations of PIs with IMiDs and steroids, represent the backbone of myeloma treatment with the addition of new drugs. The concept of immunochemotherapy, with the application of antCD38 MoAbs, currently represents the mainstream of the relapse- and first-line MM treatment [56].

In the front-line therapy settings, bortezomib-based combinations predominate [5,50]. Triplet bortezomib-based combinations, optimally including IMiDs of the first or second generation, or quadruplets with anti-CD38 MoAb, daratumumab, became the new standard of care in newly diagnosed ASCT eligible patients. Preferable first-line treatment for ASCT ineligible patients would also be daratumumab-based combos [5].

In addition to the current recommendations of maintenance therapy with lenalidomide following HDT+ASCT, there is the established concept of the long-term, continuous treatment until the progression of the disease in transplant-ineligible patients has ceased [56,57].

At relapse, in the view of clonal evolution during the course of the disease, as well as the evolving character of TME, with the goal of individual personalised treatment approach, control re-staging may be considered, particularly in patients initially characterised with standard risk features [58]. The treatment of choice in relapse, based on the duration of remission, the type of previous treatment and its toxicity, and eligibility for salvage HDT+ASCT, also incorporates control comorbidity and prognostic scores, including evolving CAs and TME [59,60,61]. 

## 4. Perspectives—Immune Oncology Treatment Options

The importance of immunotherapeutic approaches in MM was rediscovered with the introduction of IMiDs, followed by powerful modalities, such as targeted MoAbs, chimeric antigen receptor T (CAR-T) cells, antibody–drug conjugates (ADC), or bispecific T-cell engagers (BiTEs). Similar to the observed development of resistance on chemotherapy, there are three major mechanisms of TME mediated immune evasion, resulting in the escape of MM cells from immunotherapy: immune suppression, exhaustion, and resistance [62]. Immunosuppression of T- and NK cells is based on activities of Tregs, as well as regulatory B cells (Bregs), MDSCs, macrophages, dysfunctional dendritic cells, MSCs, and osteoclasts. The TME-mediated immune exhaustion is caused by pronounced expression of immune checkpoints on T- and NK cells and their ligands on MM cells, such as PD1/PDL-1, or T cell immunoglobulin and tyrosine-based inhibitory motif (TIGIT) domains. During the course of the disease, immune resistance can be developed against cytotoxic mechanisms of immune effector cells, soluble factors, or direct contact between MSCs and MM cells [63,64,65].

Adaptive cell therapy with CAR-Ts has been developed to induce autologous T-cell-mediated MM cytotoxicity by direct binding to the antigen on MM cells, followed by activation of T cells, consequently overcoming immunosuppressive TME mechanisms [66,67]. Currently, the most promising results are obtained with CAR-Ts targeting B-cell maturation antigen (BCMA) on MM cells [68]. 

In addition, BCMA is also an optimal target for antibody–drug conjugates (ADC) consisting of monoclonal antibodies and cytotoxic drugs, resulting in the internalisation of cytotoxic components and death of MM cells [69,70].

BiTEs represent engineered molecules, targeting simultaneously a cell-surface molecule on T cells (CD3) and antigen on MM cells, consequently inducing T cell response and killing of MM cells. Similar to CAR-T cells and ADCs, BCMA currently represents the most promising target. In comparison to the CAR-Ts, BiTEs are characterised by relatively simple production, allowing immediate treatment [70,71,72,73]. 

The variety of these immunotherapeutic modalities characterises the ability to overcome TME immunosuppression. Further clinical investigations of efficacy and safety are needed in order to identify the most effective and best tolerated targeted immunotherapy [74]. 

## 5. Conclusions

The bone marrow microenvironment is of high importance for the treatment outcome and course of the disease in MM patients. The delicate TME interplay consisting of molecular links between MM cells and bone marrow niche represents, at the same time, possible therapeutic targets. Current treatment options such as PIs, IMiDs, MoAbs, ASCT, or bisphosphonate’s support possess the ability to interact with TME and inducing restoration of bone marrow homeostasis. Future perspectives indicate optimisation of various types of immunotherapy (CAR-Ts, ADCs, BiTEs). The optimal treatment approach should be adjusted to all aspects of an individual patient’s profile including molecular genetics’ abnormalities and TME niche.
